# Positively interacting strains that co-circulate within a network structured population induce cycling epidemics of *Mycoplasma pneumoniae*

**DOI:** 10.1038/s41598-018-36325-z

**Published:** 2019-01-24

**Authors:** Xu-Sheng Zhang, Hongxin Zhao, Emilia Vynnycky, Vicki Chalker

**Affiliations:** 1 Centre for Infectious Disease Surveillance and Control, Public Health England, London, UK; 2 Medical Research Council Centre for Outbreak Analysis and Modelling, Department of Infectious Disease Epidemiology, Imperial College School of Public Health, London, UK; 3 TB Modelling Group, TB Centre, Centre for Mathematical Modelling of Infectious Diseases and Faculty of Epidemiology and Population Health, London School of Hygiene and Tropical Medicine, London, UK

## Abstract

*Mycoplasma pneumoniae* (MP) is considered a common cause of pneumonia, causing about 15–20% of adult community-acquired pneumonia (CAP) and up to 40% of cases in children. It has often been observed that MP epidemics last approximately 1–2 years and occur every 3–7 years, with the dominant strains alternating between epidemics. However, the underlying mechanism by which these cycles and changes in the dominant strains occur remains unclear. The traditional models for the periodicity of MP epidemics neglected two phenomena: structured contact patterns among people and co-circulating strains of MP. We also believe that the two distinctive aspects of MP epidemics: prevalent serotype shifts among epidemics and incidence cycling of MP, are interconnected. We propose a network transmission model that assumes two strains of MP are transmitted within a network structured population and they can interact as secondary infections with primary infections. Our studies show that multiple strains that co-circulate within a network structured population and interact positively generate the observed patterns of recurrent epidemics of MP. Hence our study provides a possible mechanism for the cycling epidemics of MP, and could provide useful information for future vaccine design and vaccine evaluation/monitoring processes.

## Introduction

*Mycoplasma pneumoniae* (MP) is an “atypical” bacterium that causes acute respiratory infection in humans of all ages. MP is considered a common cause of pneumonia: MP causes about 15–20% adult community-acquired pneumonia (CAP) and up to 40% cases in children; however, not every infected patient actually develops pneumonia^1–3^. MP infection generally tends to occur more frequently during the summer and autumn months when other respiratory pathogens are less prevalent; but the disease incidence does not appear to be related to season or geography^4,5^. For example, we also notice that MP infections have been observed to occur more frequently in winter months in England and Wales^6,7^. Epidemics of MP tend to occur every 3–7 years in the general population^6–8^. Analysis of laboratory reports of MP infections in England and Wales from 1975 to 2009^9^ has indicated that these epidemics last on average 18 months occurring at approximately four yearly intervals. MP is a polymorphic pathogen^10,11^: for example, Chalker *et al*.^6^ identified eleven strain types circulating in England and Wales during October 2010 to January 2011. MP strains can be differentiated based on differences in the P1 adhesin gene or in the number of repetitive sequences at a given genomic locus using multilocus variable number tandem repeat analysis (MLVA)^12,13^. Kenri *et al*.^14^ noticed that more than one serotype of MP were circulating within Japanese populations. Kogoj *et al*.^15^ observed a shift in the dominant MP strain between two epidemics that occurred in Slovenia in 2006 and 2016. Multiple strains of MP and their co-circulation were also observed in other countries (e.g.^7,16–19^). Although there are many different isolates and strains, analysis of repetitive elements distributed in variable size and sequence over the genome of MP strains suggested two main types: P1 type 1 and P1 type 2^14,16,20,21^.

Humans are the sole reservoir of MP and transmission requires close contact. Outbreaks typically occur within closed populations, such as in schools, military premises and prisons. Airborne spread of aerosols and, potentially, indirect contact with contaminated items, may contribute to transmission. The transmissibility of an infectious agent can be estimated by calculating the basic reproduction number (*R*_0_), which is defined as the mean number of secondary infectious cases generated by one primary infectious case introduced into a totally susceptible population^22^. Using seroprevalence data from a western population, Nguipdop-Djomo *et al*.^9^ estimated *R*_0_ of MP to be 1.7 (95% confidence interval (CI) 1.6–1.9), indicating low transmissibility. The incubation period of MP averages 2 to 3 weeks. The duration of infectiousness is unclear and is commonly estimated to be up to 3 weeks from onset of illness^23^. Immunity occurs post infection, but later re-infection with different subtypes is recognized, suggesting the immunity is not lifelong and no strong cross protection between different subtypes^21,24,25^. The duration of immunity ranges from 2 to 10 years^26^.

Seasonal forcing in transmission has been proposed as one determinant for the periodic patterns in other infectious diseases^27^; however, Omori *et al*.^28^ found that the seasonal forcing that occurs annually cannot generate the multi-year periodicity of MP incidence. They^28,29^ further proposed that the certain finite delay in the progression from immunity to the susceptible may provide an explanation to the occurrence of the cyclic epidemics of MP infections. More concretely, Omori *et al*.^28^ show that “minor variation in the duration of immunity at the population level must be considered essential for the MP epidemic cycle because the MP cyclic incidence pattern did not replicate without it.” As shown in Fig. 3 of ^28^, this requires that the distribution for the duration of immunity should have a variance of around 0.63. Up to now no empirical data are available for estimating the distribution of the duration of MP immunity.

The MP incidence in England and Wales has declined^7^ following the widespread use of macrolides antibiotics since introduction in the late 1990s’^30^. Due to the emergence of macrolide-resistant strains, MP infections are of increasing public health interest^31,32^. An understanding of the mechanisms by which recurrent epidemics of MP infection occur is urgently needed to enable control of future epidemics.

The two distinctive aspects of the MP epidemics: the prevalent serotype shifts among epidemics^7,14,15,19,33^ and cycling of MP incidence may be interconnected. This has been proposed before. It was argued^16,17^ that MP epidemics arise due to a change in the two main P1 types and variants of P1 sequences. Increased incidence of MP infection was observed to correlate with co-circulation of multiple strains within the population of England and Wales^6^. It was further speculated^7^ that dominant strain shift may be the cause of recurrent MP epidemics in view of the presence of multiple strains in observed increases of MP infection. Despite a lack of current data (due to limited focus on MP internationally and poor tools for detection and simultaneous strain discrimination) we speculate that serotype interactions such as synergistic associations and competition, in addition to the cross-immunity of differing P1 types, exist and play a possible role in the recurrent epidemics of MP infections. Previous transmission dynamics models (see the review of ^28^) neglected the following phenomena: co-circulation within human populations of multiple strains of MP and network structural contact patterns among people. Infection transmission depends on the contact rate as well as whom each individual contacts. Recent studies^34^ showed that people do not mix randomly. For example, contact patterns between people may display the characteristics of scale-free networks^35^ or small-world networks^36^. An important parameter of a network is its degree, defined as the number of other individuals to which one is connected. A well-mixed network (i.e., the loose network) will have a high average degree while a less mixed network should have a small average. Realistic networks of contacts that are relevant to infectious diseases usually have a small average degree^37^. On the contrary, the assumption of random mixing, in which every person is equally likely to contact any other person within the population^27,38^, results in a very large degree.

**Figure 1:**
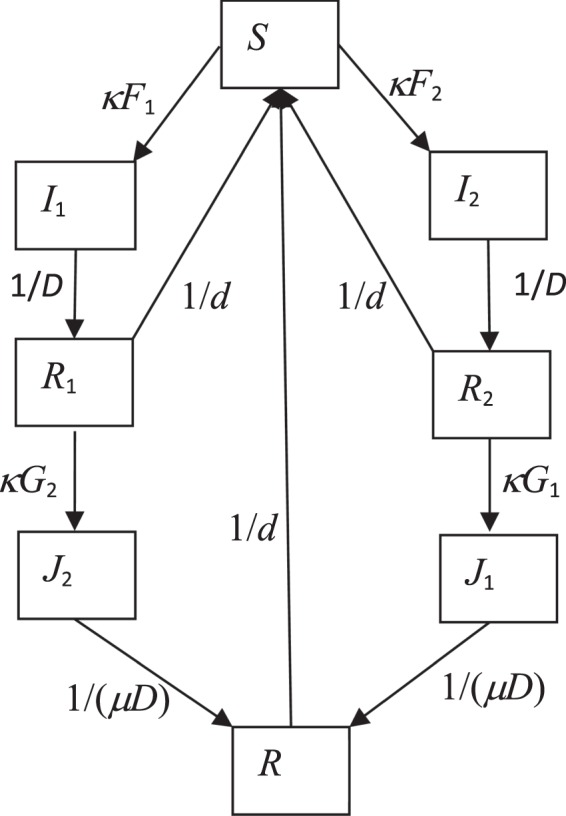
Flow chart of the two-strain SIRS epidemic model. Arrows indicate transitions. Expressions next to arrows show the *per capita* flow rate between compartments. Births and deaths are not shown. Parameter *κ* is the degree of contacts each person possesses and *μ* is the effect of primary infection strain on the duration of infection by the secondary strain. Variables *F*_1_ (*F*_2_) and *G*_1_ (*G*_2_) are forces of infection of strain 1 (strain 2) that are defined in equation (4).

Network structured models describe the transmission dynamics as in spatial transmission processes among connected groups and thus induce spatial correlation between infections. Letting infection spread on a homogeneous population with a fixed random network structure, Rozhnova and Nunes^39^ illustrate that this spatial correlation within Susceptible-Infectious-Recovered-Susceptible models assists the generation of sustained cyclical epidemics. However, strong spatial correlation (i.e., strong network structure) was needed for the cycles to persist when they just considered the transmission dynamics of a single strain in a population. Considering a two strain version of the SIRS epidemic network model^40^, the restriction on model parameters especially the degree of contacts is much relaxed. Recurrent epidemics were also predicted by models in a population which did not have a network structure, but in which people could be re-infected or co-infected with multiple strains^41^. Neither of these studies considered MP infection. In this study, we consider a two strain model as depicted in Fig. 1 and explore whether inclusion of both factors – a) competition between strains in a network-structured population and b) re-infection and interaction of secondary infection strain with primary infection strain – can explain the observed cycles in MP incidence.

## Results

We study two specific scenarios in relation to the occurrence of MP epidemic cycles. First, assuming that two strains of MP interact only through the cross-immunity during the reinfection process we sought to explore how network-structured contacts alone can help build up the characteristics of MP epidemics. Secondly we assume that the primary infection can influence the infectivity and duration of infectiousness of a secondary infection, in addition to the cross-immunity. Under this situation, we examine how these strain interactions can help generate sustained recurrent epidemics and thus relax the requirement of network contacts for the build-up of MP epidemics.

### Special situation I: network contact and cross-immunity alone

**Figure 2:**
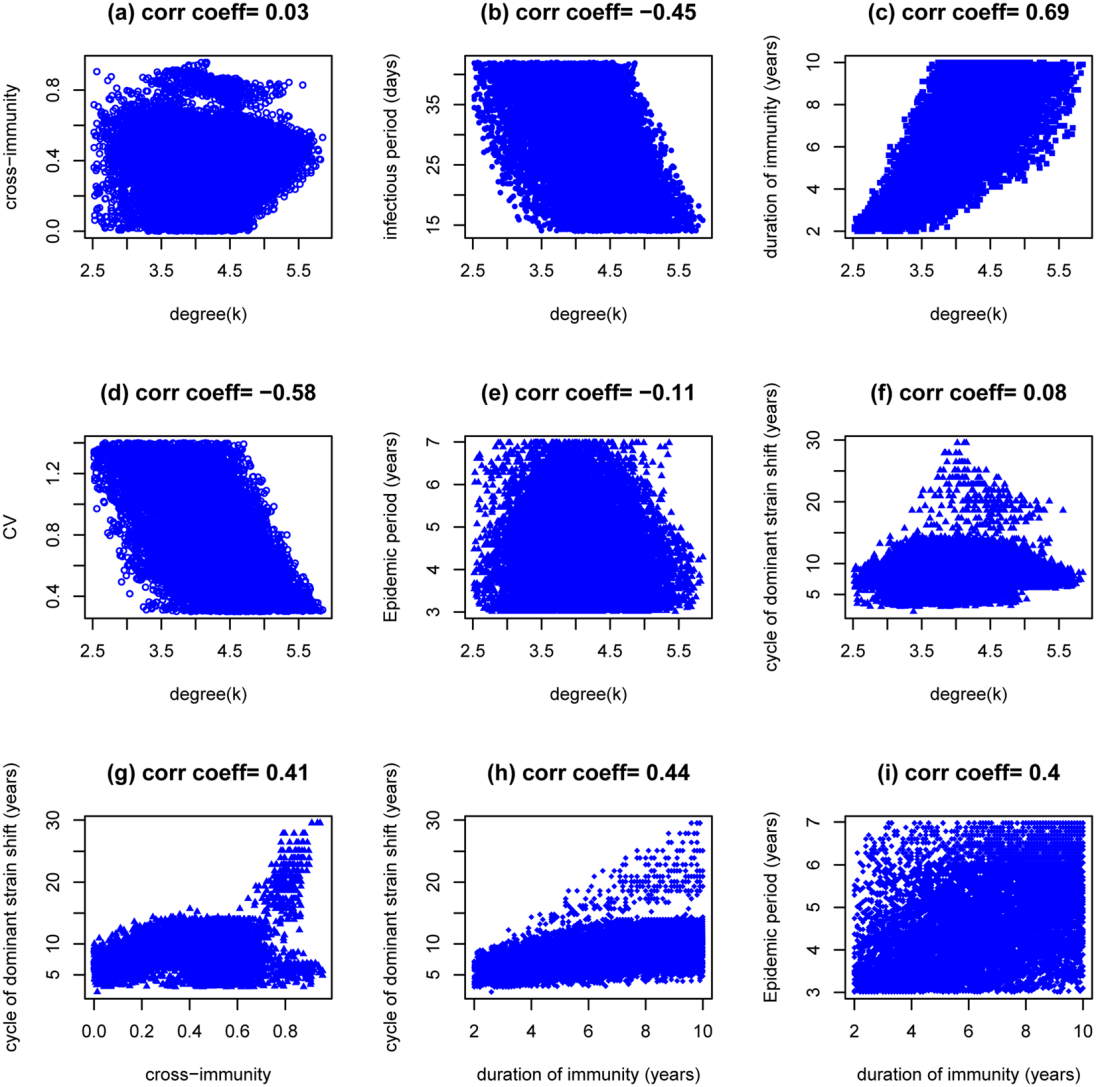
Features of LH sampling of model parameters and dynamic patterns of incidence. Here shown are dynamic patterns caused by two asynchronous strains under the condition of no interactions within the secondary infection (i.e., *ν* = *μ* = 1). The basic reproduction number *R*_0_ = 1.7 is assumed.

This is the situation where interaction parameters *ν* and *μ* are both set to 1 so strains don’t interact within secondary infections. Preliminary sampling experiments show that under this situation the model system cannot generate recurrent epidemics when contact degree (*κ*) is greater than 6.0. 150,000 combinations of model parameters were sampled with *ν* = *μ* = 1 and *κ* ranging from 2.5 to 7.0. Only 11222 combinations generate characteristically recurrent epidemics of MP that are of asynchronous strains and their features are shown in Fig. 2. Other 214 combinations generate recurrent epidemics that are of synchronous strains (see Fig. S1). The results shown in Fig. 2a illustrate that reproducing the characteristically recurrent epidemics of MP is not possible unless the contact degree (*κ*) is less than 6.0. That is, without strain interaction within the secondary infection (i.e., *ν* = *μ* = 1), it requires strong network-mediated spatial correlation (c.f.^39,40^) to enable MP epidemic cycling. When cross-immunity (*ψ*) is not extremely strong (shown in Fig. 2a), two strains asynchronously shift among epidemics. It is worth mentioning two extreme situations when *ψ* = 0 and 1. The former corresponds to the case of co-circulation of two independent strains. Figure 2a show that under this situation, recurrent epidemics of MP can be generated only when the contact degree (*κ*) is further less than 4.8. The latter corresponds to the case of single strain circulation^39,40^. Recurrent epidemics can be produced when the contact degree (*κ*) is less than 4.2 (see Fig. S1) but the two strains completely synchronise.

For the model parameters that can generate recurrent epidemics of MP, the infectious period is negatively correlated while the duration of immunity is positively correlated with the degree of contact (Fig. 2b,c). This suggests that, all other parameters being equal: within a population of a relatively large contact degree, the infectious period will need to become shorter while the duration of immunity needs to become longer for recurrent epidemics consistent with those observed to occur. Recurrent epidemics generated by a population of small contact degree (*κ* being just larger than 2.5) have a high coefficient of variation and show strong oscillations while those generated by the population of large contact degree (*κ* being just less than 6.0) have low coefficient of variation (Fig. 2d). Compared to other parameter combinations, both situations result in slightly shorter durations of recurrent epidemics and cycles of dominant strain shift (Fig. 2e,f), however. Cycles of dominant strain shift are positively associated with the presence of cross-immunity and the duration of immunity (Fig. 2g,2h), whilst durations of recurrent epidemics are insensitive to these parameters (data not shown).

**Figure 3:**
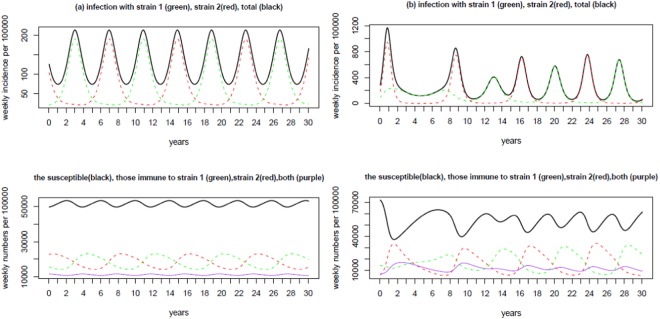
Two examples of epidemic curve from LH samples under the situation of no interaction within the secondary infection (i.e., *ν* = *μ* = 1). Panel (a) degree of contact (*κ*) = 5.69, cross-immunity (*ψ*) = 0.415, infectious period (*D*) = 17.2 days and duration of immunity (*d*) = 9.2 years, inter-epidemic period (EpiT) = 4 years, the coefficient of variance of the incidence time series (CV) = 0.51; panel (b) *κ* = 3.34, *ψ* = 0.202, *D* = 37.7 days, *d* = 4.1 years, and EpiT = 4 years, CV = 0.93. The legend is provided in the title to each figure. It is obvious that the proportion of individuals that are simultaneously immune to both strains is kept low in the two examples. The proportion of individuals immune to one strain alone is temporally highly anti-correlated with the proportion of these immune to the other strain alone, with the correlation coefficient −95% and −84% for panel (a) and panel (b) respectively.

Two examples of the predicted recurrent epidemics are demonstrated in Fig. 3: one is a regular recurrent epidemic and the other irregular. To illustrate the possible mechanisms of oscillation in incidence and the shift of the dominant strain, we also plot the changes in the susceptible individuals, and the individuals that are immune to strain 1 alone, and to strain 2 alone, and to both strains together. In Fig. 3a MP epidemics occur regularly with epidemic period of exactly 4 years and the coefficient of variance (CV) of the incidence time series is 0.51. Two strains alternate the dominancy symmetrically from one epidemic to another: when one strain is dominant the other strain remains at extremely low activities. That is, each separate epidemic is mainly caused by one strain. In Fig. 3b the duration of recurrent epidemics ranges from 3 years to 5 years with an average of four years. The average CV is 0.93, indicating a strong oscillation comparing to example shown in Fig. 3a. The epidemics also vary in the total number of infections. During each epidemic, infections can be due to either mainly one strain or two strains simultaneously.

Comparisons of the upper and bottom graphs in each panel of Fig. 3 show that infections oscillate following the changes in proportion of the population that is susceptible. The shift of the dominant strain during the oscillating epidemics is due to changes in the proportion of the population that is immune to different strains: The incidence of one strain will increase when the proportion of people immune to it is low; at the same time the incidence of the other strain will decrease because of the relatively high proportion of the population that is immune. This observation seems to support the hypothesis that a decline in immunity or an increase of the immunologically naïve population may result in the 4-year cycle of epidemic periods^6^.

### Special situation II: network contact and strain interactions via cross-immunity during re-infection and interactions within secondary infection

**Figure 4:**
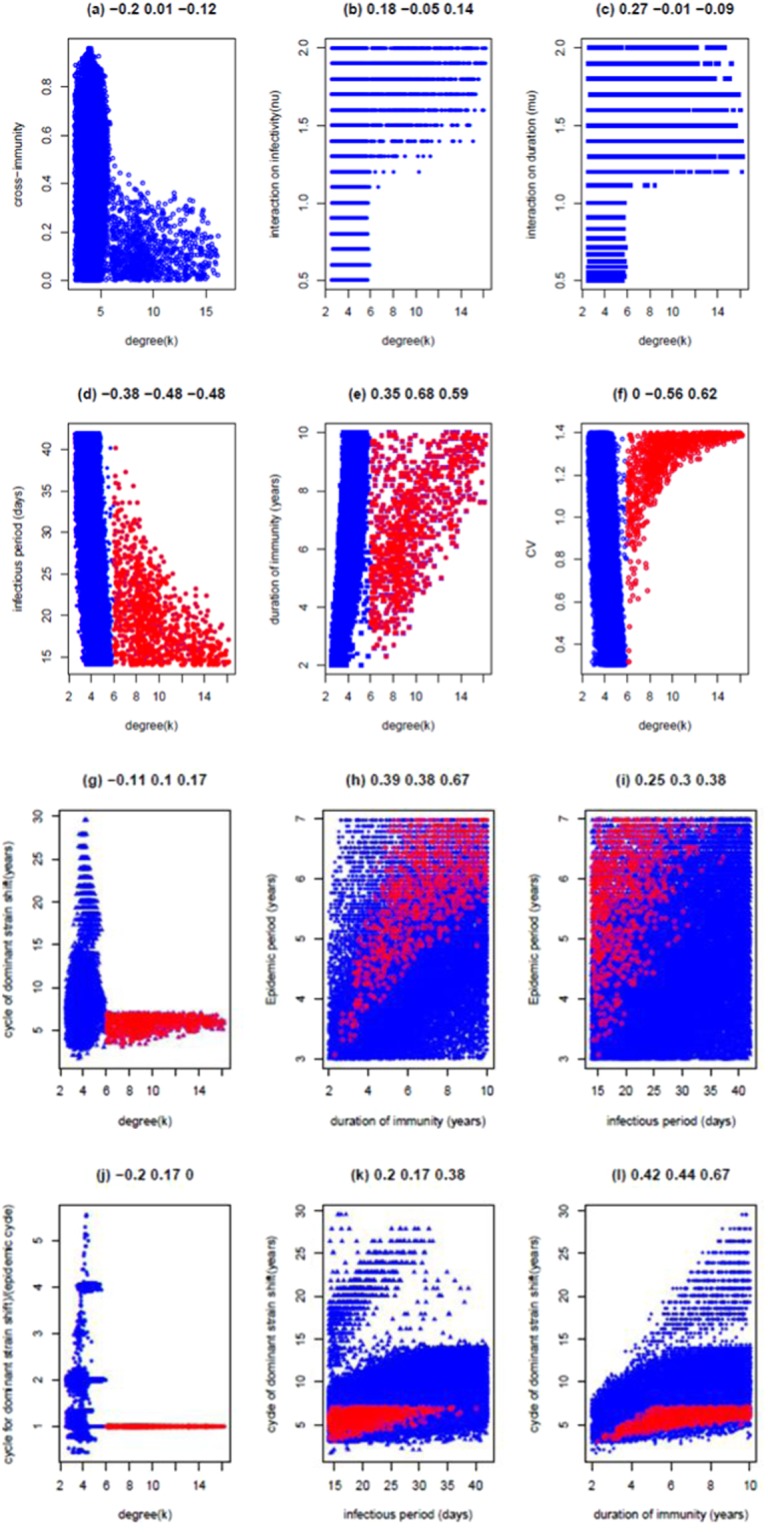
Features of LH samplings of model parameters and dynamic patterns of incidence. Here shown are dynamic patterns caused by two asynchronous strains that interact within the secondary infection. The basic reproduction number *R*_0_ = 1.7 is assumed. Panel (a) shows the maximum degree of contacts is 16.2 while panels (b) and (c) show that the critical degree for the asynchronous strains is *κ*_ac_ = 6.0. In panels (d–l) the blue points represent the parameter values when contact degrees *κ*  ≤ *κ*_ac_ and the red points for those with contact degrees *κ* > *κ*_ac_. The three values above each panel represent the correlation coefficients between the two variables for all values, the values when *κ* ≤ *κ*_ac_, and the values when *κ* > *κ*_ac_.

Preliminary sampling experiments indicate that the number of parameter combinations that can generate MP recurrent epidemics decreases quickly as the degree of contacts increases. When the degree of contacts (*κ*) exceeds 15, the combinations of model parameters for MP recurrent epidemics become extremely rare. To save the computational time, the model parameter values were sampled by dividing them into groups by the ranges of *κ*: 2.5–6, 6–8, 8–10, 10–12, 12–14, 14–16, 16–17 with respective sampling sizes 200,000, 200,000, 250,000, 250,000, 250,000, 250,000, 500,000. We obtained 23534 combinations of model parameter values that generate characteristically recurrent epidemics of MP that are of asynchronous strains; the maximum of *κ* is 16.2 (see Fig. 4a). Other 4426 combinations generate recurrent epidemics that are of synchronous strains (see Fig. S2).

The results shown in Fig. 4 indicate that compared to the above situation (I), allowing for strain interactions within secondary infections can lead to the characteristically recurrent epidemics of MP even in a population that has little network mediated spatial correlation. The maximum degree of contacts (16.2) is much larger than the maximum value of 5.9 that was required for situation I. The distributed patterns in Fig. 4a–c suggest that there is a critical threshold in the degree of contacts (denoted by *κ*_ac_ for asynchronous strain recurrent epidemics thereafter in the paper) separating the mechanisms by which recurrent epidemics consistent with those seen for MP occur. For the parameter values given in Fig. 4, *κ*_ac_ = 6.0. For populations that are of contact degree *κ* < *κ*_ac_, recurrent epidemics occur because of the spatial correlation induced by strong network structure whilst for the populations of relatively loose network structure (*κ* > *κ*_ac_), they occur because of the combination of spatial correlation and strain interactions. We refer them as mechanism 1 and mechanism 2 respectively. It is clearly shown in Fig. 4a that although cross-immunity can be any level from 0 to 1 under mechanism 1, only weak cross-immunity levels (<0.4) are required under mechanism 2.

As shown above (special situation I), when *κ* < *κ*_ac_ (mechanism 1), characteristically MP recurrent epidemics are readily generated irrespective of whether the primary infection affects the secondary infection. When strain interactions are present, complicated epidemics can be generated. Under the loose network structure (*κ* > *κ*_ac_) (mechanism 2), MP recurrent epidemics can be produced only when the primary infection enhances the infectivity (*ν* > 1) (Fig. 4b) and prolongs the infectious period (*μ* > 1) (Fig. 4c) of the secondary infection. Conversely, if strain interactions diminish the transmissibility of a secondary infection, shifts in the dominant strain in epidemics cannot occur. As in special situation I, relatively short infectious periods of MP at populations of high contact degree are required while relatively longer durations of immunity are needed to generate the recurrent MP epidemics (Fig. 4d,e). The conditions for the emergence of synchronous strains are different and are shown in Fig. S2.

Dynamic patterns including the shape of oscillations and durations of recurrent epidemics and cycle of dominant strain shift are shown in Fig. 4f–l. Figure 4f shows that the shape of the epidemic curve (i.e., CV of incidence along the time) is positively correlated with κ when *κ* > *κ*_ac_, while CV decreases with κ when *κ* ≤ *κ*_ac_. This suggests that within a looser networked population, the oscillation in incidence tends to become stronger. However, CV is not sensitive to the other model parameters (data not shown).

The cycle of dominant strain shift ranges from 3 to 30 years (Fig. 4g), which covers the observational ranges: 10–16 years^14,15^. Figure 4j indicates that when *κ* < *κ*_ac_, the cycle of dominant strain shift can be 1–6 times the duration of recurrent epidemics; when *κ* > *κ*_ac_, the cycle of dominant strain shift approximates the epidemic cycle. Both the duration of recurrent epidemics and cycle of dominant strain shift are positively associated with the duration of immunity, especially under mechanism 2 (Fig. 4h,l). Under mechanism 2, they are weakly and positively associated with the infectious period (Fig. 4i,k). Otherwise, they are insensitive to other parameters (see Fig. 4g for the relationship between cycle of dominant strain shift and degree of contacts).

**Figure 5:**
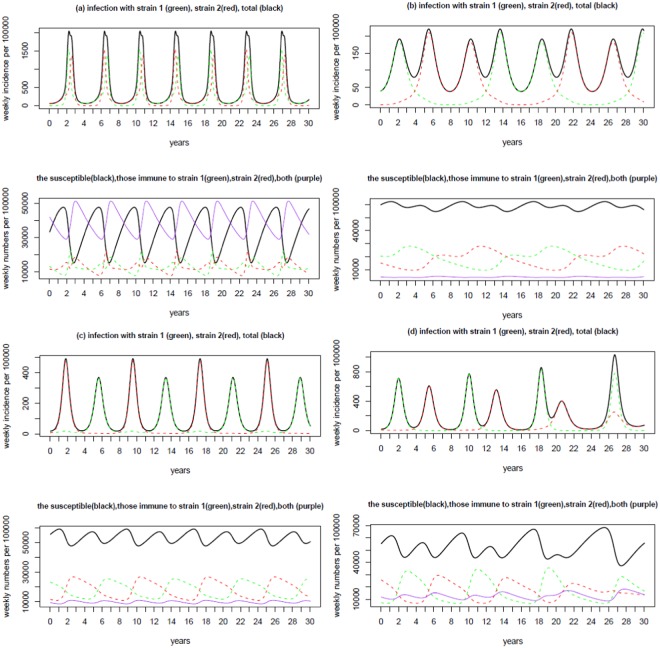
Four examples of epidemic curve from LH samples that generate MP recurrent epidemics with two asynchronous strains that interact within secondary infections. Panel (a) degree of contact (*κ*) = 8.8, cross-immunity (*ψ*) = 0.01, infectious period (*D*) = 15.4 days, duration of immunity (*d*) = 4.3 years, relative infectiousness of secondary infection (*ν*) = 1.8, relative infectious period of secondary infection (*μ*) = 1.43, and inter-epidemic period (EpiT) = 4.0 years, the coefficient of variance of the incidence time series (CV) = 1.16; (b) *κ* = 5.1, *ψ* = 0.70, *D* = 14.4 days, *d* = 8.6 years, *ν* = 1.4, *μ* = 0.59, and EpiT = 4.0 years, CV = 0.48; (c) *κ* = 5.0, *ψ* = 0.42, *D* = 14.1 days, *d* = 8.8 years, *ν* = 1.2, *μ* = 0.77, and EpiT = 4.0 years, CV = 0.90; (d) *κ* = 3.67, *ψ* = 0.19, *D* = 32.6 days, *d* = 5.4 years, *ν* = 1.6, *μ* = 0.63, and EpiT = 4.0 years, CV = 0.60. The legend is provided in the title to each figure.

Four typically recurrent epidemic examples are illustrated in Fig. 5. They show different oscillation patterns. In panel (a) two strains are of comparable activity levels with the strain that starts early dominating the epidemics; strain dominancy alternates regularly among epidemics cycle and oscillate with the same period of four years. In panel (b) two strains shift dominancy with each strain dominating two epidemics consecutively before switching strain dominancy; the two consecutive epidemics are mainly activated by the dominant strain while the other strain remains at very low activity. In panel (c) although epidemics take place regularly, recurrent epidemics consist of two different epidemics: one with high peak and narrow active period, the other with lower peak but wide active period; two strains alternate their dominancy accordingly. In panel (d) two strains alternate with irregular peaks and total incidence within each epidemic. The diverse patterns may mimic real observations in MP epidemics^7,14^.

Comparing the levels of infection and of immunity can shed light on the underlying mechanisms of recurrent epidemics. It is obvious from the Fig. 5 that the proportion of individuals that are simultaneously immune to both strains is kept low except for panel (a) where it oscillates within a wide range and anti-correlates with the proportion susceptible. The proportion of individuals immune to one strain is temporally highly anti-correlated with the proportion of these immune to the other strain, with absolute correlation coefficients >80% for panels (b), (c) and (d); while for panel (a) they are weakly correlated. This difference reflects their different levels of cross-immunity. Panels (a) and (b) show predictions obtained for a situation in which the primary infection strain increases the infectivity of secondary strain (ν > 1). In panel (a) although the dominant strain shifts between epidemics, the difference in the proportion immune or susceptible to the dominant and non-dominant strains is small. In panel (b), one strain is dominant while the other remains at a very low incidence, which continues over further epidemics even if the proportion of individuals that are immune to the strain exceeds the proportion that is immune to the other strain. The dominancy only changes when the difference in immunity to a given strain increases substantially. So under this situation, every strain dominates continuously over two epidemics before the strain dominancy switches. As found for situation I, oscillations in the infection incidence follow changes in the proportion of the population that is immune and susceptible. The change of dominant strain during the recurrent epidemics is due to the exchange in immunity to different strain: the increase in infection activity of one strain follows the relatively low immunity to the strain^6^.

**Figure 6:**
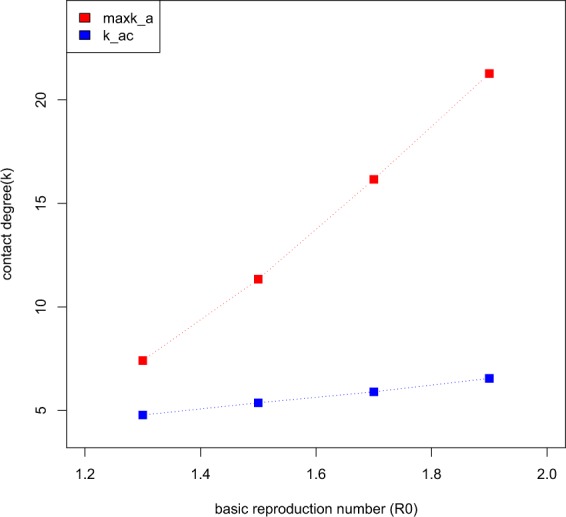
Critical threshold degrees of contacts under different transmissibility.

The findings assuming values for *R*_0_ of 1.3 and 1.5 are similar to those obtained assuming a value of 1.7, although the critical threshold value in the degree of contacts differs (see Fig. 6). Under a low value for *R*_0_ of 1.3, for example, the critical degree of contact decreases to *κ*_ac_ = 4.7 and the maximum contact degree decreases to 7.4.

## Discussion

In this study we demonstrate that spatial correlation mediated by contact network of human population and positive strain interactions within secondary infection work cooperatively to drive MP infection incidence into recurrent epidemics occurring every three to seven years with dominant strains shifting among epidemics.

Accounting for realistic host population structure in infectious disease modelling is important. It has been recognised that it is necessary to take true network contacts among human populations to explain the observed dissemination patterns of infectious disease (e.g.^42^). The results shown in Fig. 2 where no strain interaction is assumed illustrate that contact networks of degree less than 6 are required for the recurrent epidemics that occur every 3–7 years with alternation of dominant strain. A key property of a network is its degree distribution. For community, school and hospital networks, empirical studies suggest that the average degree is 6.5^37^. Other studies show higher average degrees; For example, it was estimated to be about 8.3 within social encounter networks in Great Britain^43^ and 12.5 among Hong Kong residents, a population inhabiting one of the highest density locations in the world^44^. If this empirical information reflected the true characteristics of human contact networks, then the network model without strain interaction within secondary infection could not be a candidate mechanism for MP recurrent epidemics.

Interaction between different strains during re-infection (i.e. cross-immunity) is well known and has attracted much effort to study and measure it. Once an individual is re-infected by another strain, are there any interactions between the primary infection strain and the secondary infection strain within the secondary infection? Surely recovery from primary infection will not leave immunocompetent host individuals naïve. It is theoretically reasonable to argue that the non-naïve individuals would have other changes which might in some ways alter the secondary infection by other strains (see^41^ for more general reasoning). For example, primary infection with one strain of dengue virus can enhance transmission of subsequent infection with another strain^45^. This occurs through a mechanism called “intrinsic antibody-dependent infection enhancement” and this phenomenon might be involved in many viral and bacterial infections^46^.

As to MP, it parasitizes the respiratory tract epithelium of humans, the primary infection with one strain can, for example, damage the airway^47^, which could then alter the ecological niche of the secondary infection strain. Further, the primary infections of the upper or lower respiratory tract can be followed by extrapulmonary complications^48^. As far as the transmission dynamics are concerned, the modifications in the non-naïve individuals might change the infectivity and duration of secondary infection. To our knowledge, we have not found any clear empirical data for these interactions among strains of MP, although this may reflect a lack of research into MP pathogenicity. In principle, prior exposure of an individual to a strain could have no effect or either decrease or increase the individual’s ability to clear an infection with a differing strain, with potential to increase or reduce the overall transmission. The theoretical analysis in this study shows that only positive strain interactions increase the infectiousness of a secondary infection to facilitate the generation of recurrent MP epidemics. It is expected that the experimental observations and measurement of strain interactions within secondary infections will provide vital proof to support or disprove the combination of network mediated spatial correlations and strain interactions within secondary infections as a determinant of epidemic recycling of MP.

We found that there is a positive association between the durations of recurrent epidemics and the duration of immunity (Fig. 4h). This finding is consistent with that of ^49^ but differ from that of ^28^. Further, the cycle of dominant strain shift also shows positive correlations with the duration of immunity (Fig. 4l). These positive correlations become stronger under the mechanism whereby recurrent epidemics are generated by the combination of network mediated spatial correlations and strain interactions within secondary infection. Under this mechanism, both cycles are intermediately linked with the infectious period (Fig. 4i,k). In contrast to the observations of ^27,28^, the cycle of the dominant strain shift is insensitive to cross-immunity.

Despite our simplifying assumptions, the network model of the transmission dynamics of two strains presented here remains complicated. The distributions of both infectious period and the duration of immunity are implicitly assumed to be exponential. Omori *et al*.^28^ suggested that assuming that the duration of immunity follows a distribution with a variance about 0.63, which is much smaller than that of the exponential distribution, models that assume that people mix randomly can produce the periodicity of MP recurrent epidemics. Can the reduced variation in the distribution of the duration of immunity help build up the recurrent epidemics in our network model? To see this, we construct a SI_1_I_2_R_1_R_1_R_2_R_2_J_1_J_2_RR network model by separating recovery stages into two equal parts. Therein the immunity period follows a gamma distribution of shape parameter = 2. This model has 11 nodes and is described by 65 differential equations. Nonetheless, the simulations (data not shown) show that this more complicated model does not give any noticeably different results. It is a technical challenge in our network model to generate gamma distributed duration of immunity that is comparable to that required in^28^.

We modelled human population contact structure as a static, unweighted network wherein each individual has an equal number of links with other people (i.e. regular random network), although it is recognised that in reality, the number of contacts made by each person differs between individuals, varying by age and other population groups (e.g.^43,44,50,51^). One previous study^50^ showed that the regular random network may offer a reasonable approximation to highly connected networks that approximate human contact patterns. Whilst the heterogeneity in contact networks is likely to affect the occurrence and size of outbreaks, its effect on the long-term dynamics of MP is unclear, given the low infectiousness of MP. For example, studies^43^ have postulated that, unless an infection is highly infectious, such as norovirus or some haemorrhagic fevers, the total time spent with others is more important for its transmission than the number of contacts made. This follows if, as is plausible, the duration of the contact decreases as the number of contacts made per person increases. Elucidating these issues is beyond the scope of this paper and will be studied in future work.

In this study we constrained both the interaction parameters ν and *μ*, which describe the effects of primary infection strain on the infectivity and duration of infection by secondary strain respectively, at the ranges from 0.5 to 2.0. If we had widened their ranges, requirement for the limited contact degree is further reduced. This is in agreement with the previous studies^41^: under strong strain interactions alone, epidemic cycling becomes possible even under assumptions of the homogenous mixing.

To search for the model parameter combinations that generate the recurrent epidemics of MP, Latin hypercube sampling^52^ was employed. As a statistical method for generating a near-random sample of parameter values from a multidimensional distribution, it has a short come: we do not know how many parameter combinations are adequate to guarantee that a full range of all possible outcomes are explored. This difficulty has been mentioned in Results section. Another method of sampling multi-dimensional parameters recommended by^53^ can avoid this difficulty through total sensitivity analysis^54^. This surely will merit using in the studies like ours.

In conclusion, we have illustrated that multiple strains that co-circulate within a network structured population and interact positively as secondary infections with primary infections generate the MP epidemics of 3–7 year interval and alternating dominant strains. This model supports the theory that epidemic shifts in MP may be attributed to population immunity not only to the immunogenic strain in question, but also with the influence of cross protection and other enhanced effects from the second strain type and that transmission via patient networks within the population combine to produce MP epidemic cycles. Though the strain interactions within a secondary infection are theoretically possible, currently no reliable evidence exists to suggest whether either a positive or negative strain interaction occurs. We hope this study can encourage experimental studies to detect and measure interactions between strains of MP. This will benefit our understanding of MP and provide crucial information for us to predict and thus control its recurrence.

## Models and Methods

### General structure of the model

We consider a Susceptible-Infectious-Recovered-Susceptible (SIRS) transmission dynamics model of two strains in view of the fact that isolates and strains of MP can be classified into two main types: P1 type 1 and P1 type 2^14,16,20,21^. Within the SIRS model, a population of size *N* is modelled as a network in which every individual randomly contacts a fixed number (*κ*) of other individuals, and is classified into eight compartments (Fig. 1), namely those who are susceptible to infection with any strain (S), those who are infected and infectious with strain 1 or 2 (I_1_ and I_2_), those who have recovered from infection with a given strain and are susceptible to infection with the other strain (R_1_ and R_2_), those who are infected and infectious with strain 1 or 2 after recovering from previous infection (J_1_ and J_2_) and those who are immune to infection with both strains (R). We refer to people in the I_1_ and I_2_ compartments as those with “primary infection”, and to people in the J_1_ and J_2_ compartments as those with “secondary infection”. Individuals are denoted by nodes and contacts between individuals by edges.

The epidemic dynamics is determined by the following transmission and transition processes. Susceptible nodes (*S*) become infected with strain *i*, *i* = {1, 2}, at rate *λ* through an edge with a node of primary infection *I*_*i*_, or at rate *νλ* through an edge with a node of secondary infection *J*_*i*_. Here parameter *λ* represents the constant transmission rate and parameter *ν* is the infectiousness of a secondary infection relative to a primary infection. Primarily infected nodes (*I*_*i*_) stay infectious on average for *D* days before becoming fully immune (*R*_*i*_) to the infecting strain *i* and partially so to the other strain. Recovered individuals (*R*_*i*_) stay immune for an average of *d* days before becoming susceptible again, or becoming secondarily infected at rate (1-*ψ*)*λ* through an edge linked with a node of infection (*I*_3*-i*_ or *J*_3*-i*_) to become secondarily infected *J*_3*-i*_, *i* = {1, 2}. Here *ψ* reflects the reduction in susceptibility due to previous exposure to the other strain (i.e., cross-immunity). Nodes of secondary infection *J*_*i*_, *i* = {1, 2} stay infectious for an average of *μD* days before becoming fully immune to all strains (i.e., *R*). Here parameter *μ* defines the effect of having experienced primary infection on the duration of the secondary infection. Nodes of *R* stay fully immune for an average of *d* days before becoming susceptible again. Therefore naïve individuals are recruited into the population through birth and loss of immunity. These transitions and transmissions are defined according to the pairs or triplets involved in the process^39,40,55^. For simplicity we ignore clustering in the network (c.f.^37,40,55^).

### Model equations

Similar to^55^, the proportions of people in eight compartments are represented by [S], [I_1_], [I_2_], [R_1_], [R_2_], [J_1_], [J_2_], and [R]. Because of the constant population size (i.e., the constant number of nodes), [S] + [I_1_] + [I_2_] + [R_1_] + [R_2_] + [J_1_] + [J_2_] + [R] = 1. There are (8 × 7)/2 = 28 heterogeneous pairs within the network in which the two nodes of a pair are of different states. The proportion of the population that is in a pair ([XY]) is defined as

1 $$[XY]\approx \frac{{n}_{XY}}{\kappa N}$$

Here *n*_XY_ is the number of pairs within the population. The number of homogenous pairs can be found from these equations for heterogeneous pairs: e.g., [*RR*] = $(1-\sum _{Y\ne R}[Y])-\sum _{X\ne R}[XR]$. The state of the model system is defined by eight integers of nodes and 28 integers of heterogeneous pairs. To focus on the impact of spatial correlation mediated by network structure and interactions between strains, two strains are simply assumed to be antigenically indistinguishable within linked patients although they can actually be different^56^.

Infection transmission occurs through pair-link and the change of pairs is determined by the triples^40^. To close the model system, the proportion, [*XYZ*], of the triple *XYZ* with node *Y* having contacts with both *X* and *Z* is approximated in terms of the proportion of pairs as in^55^,

2 $$[XYZ]\approx \frac{k-1}{k}\frac{[XY][YZ]}{[Y]}$$

The flow chart of the epidemic model is shown in Fig. 1. The model is described by a set of 28 + 7 = 35 differential equations as,

**Equations describing the time changes in 7 nodes**

3a $$\begin{array}{rcl}\frac{d}{dt}[S] & = & \tfrac{1}{L}(1-[S])+\tfrac{1}{d}([R]+[{R}_{1}]+[{R}_{2}])-\kappa ({F}_{1}+{F}_{2})\\ \frac{d}{dt}[{I}_{1}] & = & -\,(\tfrac{1}{L}+\tfrac{1}{D})[{I}_{1}]+\kappa {F}_{1}\\ \frac{d}{dt}[{I}_{2}] & = & -\,(\tfrac{1}{L}+\tfrac{1}{D})[{I}_{2}]+\kappa {F}_{2}\\ \frac{d}{dt}[{R}_{1}] & = & -\,(\tfrac{1}{L}+\tfrac{1}{d})[{R}_{1}]+\tfrac{1}{D}[{I}_{1}]-\kappa {G}_{2}\\ \frac{d}{dt}[{R}_{2}] & = & -\,(\tfrac{1}{L}+\tfrac{1}{d})[{R}_{2}]+\tfrac{1}{D}[{I}_{2}]-\kappa {G}_{1}\\ \frac{d}{dt}[{J}_{1}] & = & -\,(\tfrac{1}{L}+\tfrac{1}{\mu D})[{J}_{1}]+\kappa {G}_{1}\\ \frac{d}{dt}[{J}_{2}] & = & -\,(\tfrac{1}{L}+\tfrac{1}{\mu D})[{J}_{2}]+\kappa {G}_{2}\end{array}$$

**Equations describing the time changes of 28 pairs**

$$\begin{array}{rcl}\frac{d}{dt}[S{I}_{1}] & = & \tfrac{1}{d}([{I}_{1}R]+[{I}_{1}{R}_{2}]+[{I}_{1}{R}_{1}])-(\tfrac{1}{D}+\lambda )[S{I}_{1}]\\  &  & +(\kappa -1)\{\tfrac{([SS]-[S{I}_{1}]){F}_{1}-[S{I}_{1}]{F}_{2}}{[S]}\}+\tfrac{1}{L}([{I}_{1}]-2[S{I}_{1}])\\ \frac{d}{dt}[S{I}_{2}] & = & \tfrac{1}{d}([{I}_{2}R]+[{I}_{2}{R}_{2}]+[{I}_{2}{R}_{1}])-(\tfrac{1}{D}+\lambda )[S{I}_{2}]\\  &  & +(\kappa -1)\{\tfrac{([SS]-[S{I}_{2}]){F}_{2}-[S{I}_{2}]{F}_{1}}{[S]}\}+\tfrac{1}{L}([{I}_{2}]-2[S{I}_{2}])\\ \frac{d}{dt}[S{R}_{1}] & = & \tfrac{1}{d}([{R}_{1}R]+([{R}_{1}{R}_{1}]-[S{R}_{1}])+[{R}_{1}{R}_{2}])+\tfrac{1}{D}[S{I}_{1}]\\  &  & -(\kappa -1)\{\frac{{F}_{1}+{F}_{2}}{[S]}+\frac{{G}_{2}}{[{R}_{1}]}\}[S{R}_{1}]+\tfrac{1}{L}([{R}_{1}]-2[S{R}_{1}])\\ \frac{d}{dt}[S{R}_{2}] & = & \tfrac{1}{d}([{R}_{2}R]+([{R}_{2}{R}_{2}]-[S{R}_{2}])+[{R}_{1}{R}_{2}])+\tfrac{1}{D}[S{I}_{2}]\\  &  & -(\kappa -1)\{\frac{{F}_{1}+{F}_{2}}{[S]}+\frac{{G}_{1}}{[{R}_{2}]}\}[S{R}_{2}]+\tfrac{1}{L}([{R}_{2}]-2[S{R}_{2}])\\ \frac{d}{dt}[SR] & = & \tfrac{1}{d}(([RR]-[SR])+[{R}_{2}R]+[{R}_{1}R])+\tfrac{1}{\mu D}([S{J}_{1}]+[S{J}_{2}])\\  &  & -\frac{(\kappa -1)[SR][{F}_{1}+{F}_{2}]}{[S]}+\tfrac{1}{L}([R]-2[SR])\\ \frac{d}{dt}[S{J}_{1}] & = & \tfrac{1}{d}([R{J}_{1}]+[{R}_{2}{J}_{1}]+[{R}_{1}{J}_{1}])-(\tfrac{1}{\mu D}+\nu \lambda )[S{J}_{1}]\\  &  & -(\kappa -1)\{\frac{[S{J}_{1}]({F}_{1}+{F}_{2})}{[S]}-\frac{[S{R}_{2}]{G}_{1}}{[{R}_{2}]}\}+\tfrac{1}{L}([{J}_{1}]-2[S{J}_{1}])\\ \frac{d}{dt}[S{J}_{2}] & = & \tfrac{1}{d}([R{J}_{2}]+[{R}_{2}{J}_{2}]+[{R}_{1}{J}_{2}])-(\tfrac{1}{\mu D}+\nu \lambda )[S{J}_{2}]\\  &  & -(\kappa -1)\{\frac{[S{J}_{2}]({F}_{1}+{F}_{2})}{[S]}-\frac{[S{R}_{1}]{G}_{2}}{[{R}_{1}]}\}+\tfrac{1}{L}([{J}_{2}]-2[S{J}_{2}])\\ \frac{d}{dt}[{I}_{1}{I}_{2}] & = & -\,2(\tfrac{1}{L}+\tfrac{1}{D})[{I}_{1}{I}_{2}]+(\kappa -1)\{\frac{[S{I}_{2}]{F}_{1}}{[S]}+\frac{[S{I}_{1}]{F}_{2}}{[S]}\}\\ \frac{d}{dt}[{I}_{1}{R}_{1}] & = & -\,(\tfrac{2}{L}+\tfrac{1}{d})[{I}_{1}{R}_{1}]+\tfrac{1}{D}([{I}_{1}{I}_{1}]-[{I}_{1}{R}_{1}])+(\kappa -1)\{\frac{[S{R}_{1}]{F}_{1}}{[S]}-[{I}_{1}{R}_{1}]\frac{{G}_{2}}{[{R}_{1}]}\}\\ \frac{dp}{dt}[{I}_{1}{R}_{2}] & = & -\,\{\tfrac{2}{L}+\tfrac{1}{d}+\tfrac{1}{D}+\lambda (1-\psi )\}[{I}_{1}{R}_{2}]+\tfrac{1}{D}[{I}_{1}{I}_{2}]+(\kappa -1)\{\frac{[S{R}_{2}]{F}_{1}}{[S]}-[{I}_{1}{R}_{2}]\frac{{G}_{1}}{[{R}_{2}]}\}\\ \frac{d}{dt}[{I}_{1}R] & = & -\,(\tfrac{2}{L}+\tfrac{1}{D}+\tfrac{1}{d})[{I}_{1}R]+\tfrac{1}{\mu D}([{I}_{1}{J}_{1}]+[{I}_{1}{J}_{2}])+(\kappa -1)\frac{[SR]{F}_{1}}{[S]}\\ \frac{d}{dt}[{I}_{1}{J}_{1}] & = & -\,(\tfrac{2}{L}+\tfrac{1}{D}+\tfrac{1}{\mu D})[{I}_{1}{J}_{1}]+\nu \lambda [S{J}_{1}]+\lambda (1-\psi )[{I}_{1}{R}_{2}]+(\kappa -1)\{\frac{[S{J}_{1}]{F}_{1}}{[S]}+\frac{[{I}_{1}{R}_{2}]{G}_{1}}{[{R}_{2}]}\}\\ \frac{d}{dt}[{I}_{1}{J}_{2}] & = & -\,(\tfrac{2}{L}+\tfrac{1}{D}+\tfrac{1}{\mu D})[{I}_{1}{J}_{2}]+(\kappa -1)\{\frac{[S{J}_{2}]{F}_{1}}{[S]}+\frac{[{I}_{1}{R}_{1}]{G}_{2}}{[{R}_{1}]}\}\\ \frac{d}{dt}[{I}_{2}{R}_{{\rm{1}}}] & = & -\,(\tfrac{2}{L}+\tfrac{1}{D}+\tfrac{1}{d}+\lambda (1-\psi ))[{I}_{2}{R}_{{\rm{1}}}]+\tfrac{1}{D}[{I}_{{\rm{1}}}{I}_{2}]+(\kappa -1)\{\frac{[S{R}_{{\rm{1}}}]{F}_{2}}{[S]}-[{I}_{2}{R}_{{\rm{1}}}]\frac{{G}_{2}}{[{R}_{{\rm{1}}}]})\}\\ \frac{d}{dt}[{I}_{2}{R}_{{\rm{2}}}] & = & -\,(\tfrac{2}{L}+\tfrac{1}{d})[{I}_{2}{R}_{{\rm{2}}}]+\tfrac{1}{D}([{I}_{2}{I}_{{\rm{2}}}]-[{I}_{2}{R}_{2}])+(\kappa -1)\{\frac{[S{R}_{2}]{F}_{2}}{[S]}-[{I}_{2}{R}_{2}]\frac{{G}_{1}}{[{R}_{2}]}\}\end{array}$$

3b $$\begin{array}{rcl}\frac{d}{dt}[{I}_{2}R] & = & -\,(\tfrac{2}{L}+\tfrac{1}{D}+\tfrac{1}{d})[{I}_{2}R]+\tfrac{1}{\mu D}([{I}_{2}{J}_{{\rm{1}}}]+[{I}_{2}{J}_{{\rm{2}}}])+(\kappa -1)\frac{[SR]{F}_{2}}{[S]}\\ \frac{d}{dt}[{I}_{2}{J}_{{\rm{1}}}] & = & -\,(\tfrac{2}{L}+\tfrac{1}{D}+\tfrac{1}{\mu D})[{I}_{2}{J}_{{\rm{1}}}]+(\kappa -1){}\{\frac{[S{J}_{{\rm{1}}}]{F}_{2}}{[S]}+\frac{[{I}_{2}{R}_{{\rm{2}}}]{G}_{1}}{[{R}_{2}]}\}\\ \frac{d}{dt}[{I}_{2}{J}_{2}] & = & -\,(\tfrac{2}{L}+\tfrac{1}{D}+\tfrac{1}{\mu D})[{I}_{2}{J}_{2}]+\nu \lambda [S{J}_{2}]+\lambda (1-\psi )[{I}_{2}{R}_{1}]+(\kappa -1)\{\frac{[S{J}_{2}]{F}_{2}}{[S]}+\frac{[{I}_{2}{R}_{1}]{G}_{2}}{[{R}_{1}]}\}\\ \frac{d}{dt}[{R}_{1}{R}_{2}] & = & -\,2(\tfrac{1}{L}+\tfrac{1}{d})[{R}_{1}{R}_{2}]+\tfrac{1}{D}([{I}_{1}{R}_{2}]+[{I}_{2}{R}_{1}])-(\kappa -1)[{R}_{1}{R}_{2}]\{\frac{{G}_{2}}{[{R}_{1}]}+\frac{{G}_{1}}{[{R}_{2}]}\}\\ \frac{d}{dt}[{R}_{1}R] & = & -\,2(\tfrac{1}{L}+\tfrac{1}{d})[{R}_{1}R]+\tfrac{1}{D}[{I}_{1}R]+\tfrac{1}{\mu D}([{R}_{1}{J}_{1}]+[{R}_{1}{J}_{2}])-\frac{(\kappa -1)[{R}_{1}R]{G}_{2}}{[{R}_{1}]}\\ \frac{d}{dt}[{R}_{1}{J}_{1}] & = & -\,(\tfrac{2}{L}+\tfrac{1}{d}+\tfrac{1}{\mu D})[{R}_{1}{J}_{1}]+\tfrac{1}{D}[{I}_{1}{J}_{1}]-(\kappa -1)(\frac{[{R}_{1}{J}_{1}]{G}_{2}}{[{R}_{1}]}-\frac{[{R}_{1}{R}_{2}]{G}_{1}}{[{R}_{2}]})\\ \frac{d}{dt}[{R}_{1}{J}_{2}] & = & -\,\{\tfrac{2}{L}+\tfrac{1}{d}+\tfrac{1}{\mu D}+\nu (1-\psi )\lambda \}[{R}_{1}{J}_{2}]+\tfrac{1}{D}[{I}_{1}{J}_{2}]+\frac{(\kappa -1)([{R}_{1}{R}_{1}]-[{R}_{1}{J}_{2}]){G}_{2}}{[{R}_{1}]}\\ \frac{d}{dt}[{R}_{2}R] & = & -\,2(\tfrac{1}{L}+\tfrac{1}{d})[{R}_{2}R]+\tfrac{1}{D}[{I}_{2}R]+\tfrac{1}{\mu D}([{R}_{2}{J}_{1}]+[{R}_{2}{J}_{2}])-\frac{(\kappa -1)[{R}_{2}R]{G}_{1}}{[R{}_{2}]}\\ \frac{d}{dt}[{R}_{2}{J}_{1}] & = & -\,\{\tfrac{2}{L}+\tfrac{1}{d}+\tfrac{1}{\mu D}+\nu (1-\psi )\lambda \}[{R}_{2}{J}_{1}]+\tfrac{1}{D}[{I}_{2}{J}_{1}]+\frac{(\kappa -1)([{R}_{2}{R}_{2}]-[{R}_{2}{J}_{1}]){G}_{1}}{[{R}_{2}]}\\ \frac{d}{dt}[{R}_{2}{J}_{2}] & = & -\,(\tfrac{2}{L}+\tfrac{1}{d}+\tfrac{1}{\mu D})[{R}_{2}{J}_{2}]+\tfrac{1}{D}[{I}_{2}{J}_{2}]+(\kappa -1)\{\frac{[{R}_{1}{R}_{2}]{G}_{2}}{[{R}_{1}]}-\frac{[{R}_{2}{J}_{2}]{G}_{1}}{[{R}_{2}]}\}\\ \frac{d}{dt}[R{J}_{1}] & = & -\,(\tfrac{2}{L}+\tfrac{1}{d}+\tfrac{1}{\mu D})[R{J}_{1}]+\tfrac{1}{\mu D}([{J}_{1}{J}_{1}]+[{J}_{1}{J}_{2}])+\frac{(\kappa -1)[{R}_{2}R]{G}_{1}}{[{R}_{2}]}\\ \frac{d}{dt}[R{J}_{2}] & = & -\,(\tfrac{2}{L}+\tfrac{1}{d}+\tfrac{1}{\mu D})[R{J}_{2}]+\tfrac{1}{\mu D}([{J}_{2}{J}_{2}]+[{J}_{1}{J}_{2}])+\frac{(\kappa -1)[{R}_{1}R]{G}_{2}}{[{R}_{1}]}\\ \frac{d}{dt}[{J}_{1}{J}_{2}] & = & -\,2(\tfrac{1}{L}+\tfrac{1}{\mu D})[{J}_{1}{J}_{2}]+(\kappa -1)\{\frac{[{R}_{2}{J}_{2}]{G}_{1}}{[{R}_{2}]}+\frac{[{R}_{1}{J}_{1}]{G}_{2}}{[{R}_{1}]}\}\end{array}$$

In the above equations, the different forces of infection are

4 $$\begin{array}{rcl}{\rm{Strain}}\,{\rm{1}}\,{\rm{infects}}\,S\,{\rm{individuals}}:{F}_{1} & = & \lambda ([S{I}_{1}]+\nu [S{J}_{1}])\\ {\rm{Strain}}\,{\rm{2}}\,{\rm{infects}}\,S\,{\rm{individuals}}:{F}_{2} & = & \lambda ([S{I}_{2}]+\nu [S{J}_{2}])\\ {\rm{Strain}}\,{\rm{1}}\,{\rm{infects}}\,{R}_{{\rm{2}}}\,{\rm{individuals}}:{G}_{{\rm{1}}} & = & \lambda (1-\psi )([{I}_{1}{R}_{2}]+\nu [{J}_{1}{R}_{2}])\\ {\rm{Strain}}\,{\rm{2}}\,{\rm{infects}}\,{R}_{{\rm{1}}}\,{\rm{individuals}}:{G}_{{\rm{2}}} & = & \lambda (1-\psi )([{I}_{2}{R}_{1}]+\nu [{R}_{1}{J}_{2}])\end{array}$$

The model parameters are defined in Table 1. Compared with the model presented in^40^, here we introduce two interaction parameters to define the effects of experiencing primary infection on infectivity (*ν*) and the duration (*μ*) of a secondary infection. The complexity of the two strain network dynamics allows us to investigate the combined effects of strain interactions (cross-immunity during re-infection and effects of the primary infection on a secondary infection) on dynamic patterns of endemic infectious diseases, along with spatial competition embedded within the random network. Ignoring the stochasticity due to the limited size of population, here we focus on these by considering an infinitely large population (i.e., *N* → ∞).

**Table 1:** Model parameters.

parameter	Definition	Values or ranges	source
*L*	Average life span	70*365 days	^57^
D	Infectious period of a single infection	14–42 days	^23^
d	Duration of immunity	2–10 years	^26^
*R*_0_	Basic reproduction number	1.7	^9^
*λ*	Transmission rate of single infection	$\lambda =\frac{{R}_{0}(d+1)}{D[d(k-2)+(\kappa -1)]}$	^55^
*ψ*	Reduction in susceptibility to a secondary infection, resulting from a primary infection (“cross-immunity”)	0.0–1.0	–
*ν*	Relative infectiousness of a secondary infection, compared to a primary infection.	0.5–2.0	Negative (<1) and positive (>1) effects
*μ*	Factor by which the duration of a secondary infection differs from that of a primary infection.	0.5–2.0	Negative (<1) and positive (>1) effects
κ	Degree of contact network: number of people with whom one person contacts.	2.5–25.0	assumed

## Methods

We used Latin hypercube sampling^52^ to identify parameter values which led to model predictions of cycles in incidence which were consistent with those observed. The values of the following parameters were sampled within the ranges listed in Table 1: infectious period (*D*), duration of immunity (*d*), degree of contact network (*κ*), cross-immunity (*ψ*), effects of primary infection on infectivity (*ν*) and duration (*μ*) of a secondary infection. This was done with the function randomLHS of the package lhs in R computing language^58^.

The basic reproduction number (*R*_0_) was fixed at 1.7 as estimated by^9^. When estimating *R*_0_, Nguipdop-Djomo *et al*.^9^ assumed random mixing among individuals and didn’t account for a network structured population. For a network structured population, the random mixing assumption will give rise to an over-estimate of *R*_0_ (Fig. 2 of ^55^), so we also consider this effect by assuming that *R*_0_ = 1.5 and 1.3 for MP. The total number of infections in our model includes both asymptomatic and symptomatic infections. Since MP may affect all age groups (e.g.^6,7,25^), we consider the situation in which the life span is 70 years, the worldwide average life expectancy according to the world fact book^57^.

A value for the interaction parameters *ν* and *μ* of 1.0 implies that there is no interaction on the 2^nd^ infection from the primary infection (This is a special situation considered in^40^). If they are less than 1.0, it means the interactions diminish the relevant processes. On the contrary, if they are larger than 1.0, they enhance the processes. The ranges listed in Table 1 allow both increasing and decreasing effects to be selected. To constrain the interactions within biologically reasonable limits, we allow both *ν* and *μ* to vary from 0.5 to 2.0. To consider the effect of network-mediated spatial correlation, the contact degree (*κ*) is allowed to vary from 2.5 to 25.

The Runge-Kutta fourth order method was used to solve the model equations (3–4). As our dynamic system is deterministic, there is one dynamical time series under each set of parameter values. For each time series in which infection persists, weekly rates of new infections with each strain are recorded: the first 800 years were discarded and 200 years were used for analysis. To monitor the time series data and calculate the inter-epidemic periods if periodic changes in the incidence of both strains and total number of infections occur, the spectrum function in the R computing language^58^ is employed. The inter-epidemic period (or the duration of epidemic cycle) will be denoted by EpiT. Following^28^, the coefficient of variance (CV) of the incidence time series was used to define the shape of epidemic curve and the strength of oscillation in infections over time. It was showed^14^ that CV in Japan MP epidemics 1982 to 1990 is about 0.7. In view of this, we regard the epidemics that possess the following characteristics as reasonable approximates to what has been empirically observed in MP epidemics: 0.3 ≤ CV ≤ 1.4 and 3 ≤ EpiT ≤ 7 years, along with dominant strain changing among epidemics. In the main text we refer to this as the “characteristically recurrent epidemics of MP”.

## Electronic supplementary material

Supplementary Information
