# Supplementary material for: Positively interacting strains that co-circulate within a network structured population induce cycling epidemics of Mycoplasma pneumoniae
Source: Sci Rep. 2019 Jan 24;9:541. doi: 10.1038/s41598-018-36325-z (PMC6345813; doi:10.1038/s41598-018-36325-z)
Supplement: Supplementary file 1 — Supplementary Information [file 41598_2018_36325_MOESM1_ESM.pdf]

Positively interacting strains that co-circulate within a network structured population induce cycling epidemics of *Mycoplasma pneumoniae*

Xu-Sheng Zhang[1,2*], Hongxin Zhao[1], Emilia Vynnycky[1,3], Vicki Chalker[1]

[1]Centre for Infectious Disease Surveillance and Control, Public Health England, London, UK
[2]Medical Research Council Centre for Outbreak Analysis and Modelling, Department of Infectious Disease Epidemiology, Imperial College School of Public Health, London, UK
[3]TB Modelling Group, TB Centre, Centre for Mathematical Modelling of Infectious Diseases and Faculty of Epidemiology and Population Health, London School of Hygiene and Tropical Medicine, London, UK.

Supporting Information

The features of recurrent epidemics of period 3 to 7 years caused by two synchronous strains

Here we consider all the combinations of model parameter values that generate the recurrent epidemics of period 3 to 7 years caused by two synchronous strains so that both strains synchronize with the total incidence.

Under the situations where there is no strain interaction except the cross-immunity during re-infection, Supplementary Figure S1 shows that synchronous strains emerge when there are strong cross-immunity (>0.84) and strong spatial correlation between strains due to strong contact network (κ <4.1) (Panel A). As κ increases (i.e., network structure becomes weak), infectious period decreases while immunity period increases (Panels B and C). The duration of recurrent epidemics is, albeit being independent of degree of contacts (Panel F),

proportional to both infectious period and duration of immunity (Panel G and H). CV is insensitive to these parameters (data not shown).

When including strain interactions within secondary infections, Supplementary Figure S2 shows the features of model parameters for synchronous strains. There is a critical degree for synchronous strains: κ_{sc} = 4.1 (panels B and C). Panel A) shows that when $\kappa \leq \kappa_{sc}$, synchronous strains emerge if there is a strong cross-immunity (ψ>0.78); while if $\kappa > \kappa_{sc}$ where enhanced interactions (ν>1 and μ>1) are needed to generate oscillation in incidence, it requires weak cross-immunity (ψ<0.4) to have synchronous strains. Panels F and G show that the duration of recurrent epidemics is proportional to both duration of immunity and infection period. For the recurrent epidemics caused by asynchronous strains shown in Figure 4, there is a maximum degree of contacts within the population. In contrast to this, we found that there is no such maximum degree required for the recurrent epidemics caused by synchronous strains (at least to the largest of sampled values listed in Table 1).

For a low reproduction number (R_0=1.3), the features of model parameters are similar to the situation of R_0=1.7 except that the critical threshold degree decreases to κ_{sc} = 3.6.

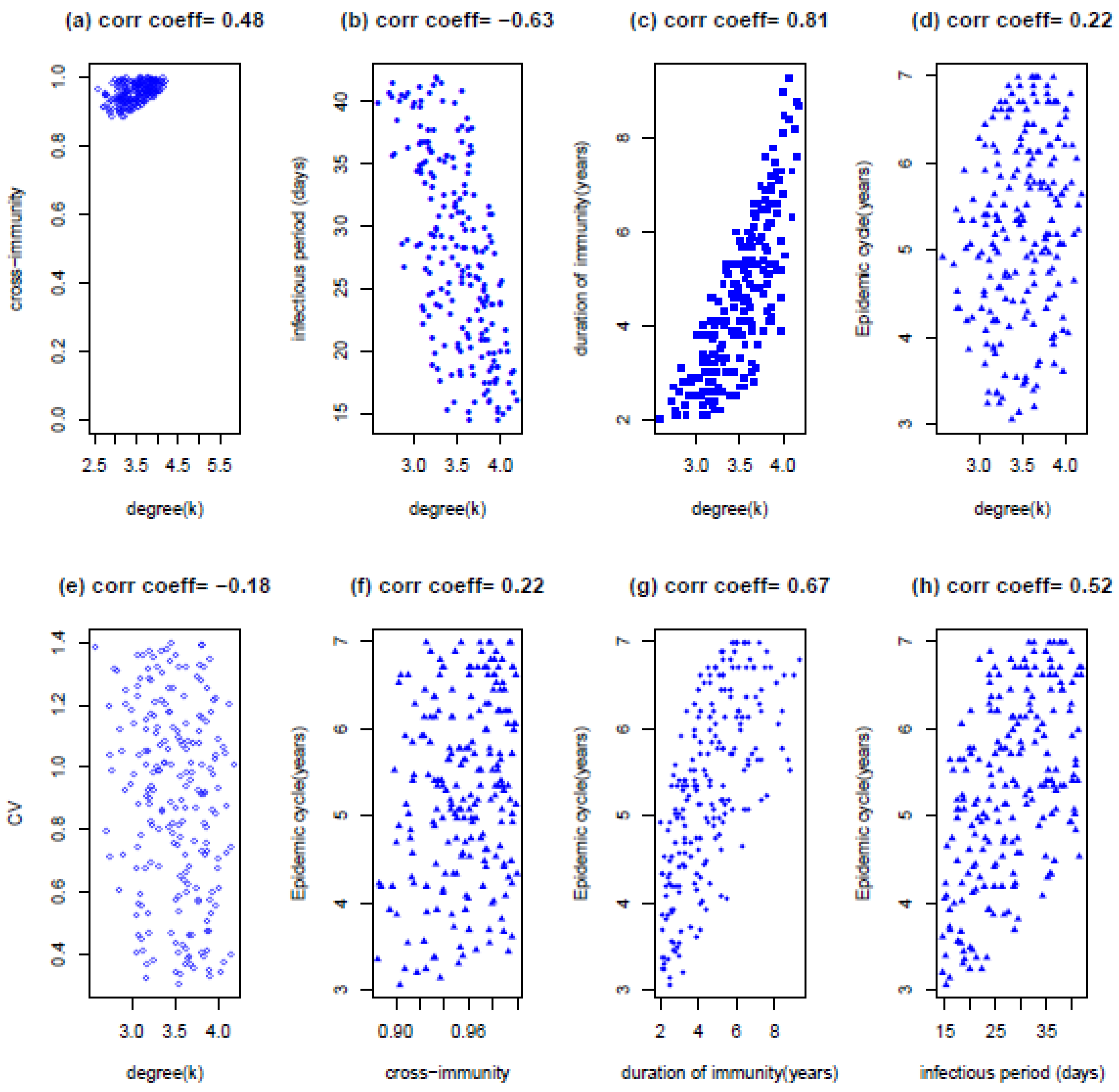

Supplementary Figure S1 Features of LH sampling of model parameters that generate recurrent epidemics by two synchronous strains without interactions within the secondary infection (i.e., $\nu=\mu=1$). An average life span of 70 years and basic reproduction number R_0 =1.7 are assumed. Among 150,000 LHS samples with $\nu=\mu=1$ and κ ranging from 2.5 to 7, 214 samples generate characteristically recurrent epidemics of MP, which are caused by synchronous strains. A) Relationship between cross-immunity (ψ) and contact degree (κ); B) Relationship between infectious period and κ; C) Relationship between duration of immunity and κ; D) dependence of epidemic period on κ, E) dependence of the shape of epidemic curve (CV) on κ, F) Relationship between period of epidemics and cross-immunity; G) Relationship between period of epidemics and immunity period, and H) Relationship between period of epidemics and infectious period.

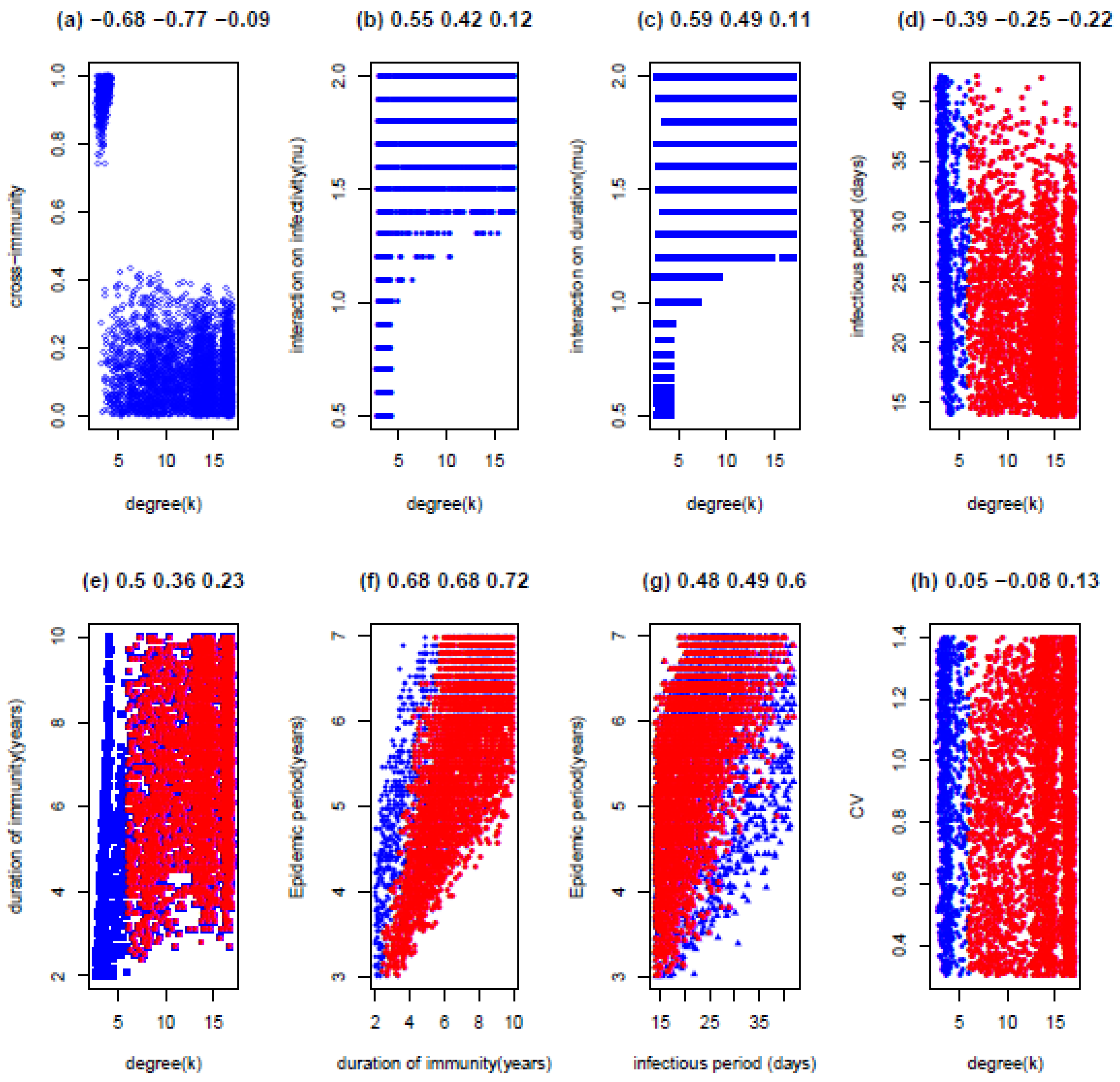

Supplementary Figure S2 Features of LH sampling of model parameters that generate recurrent epidemics by synchronous strains with interactions within the secondary infection. An average life span of 70 years and basic reproduction number R_0=1.7 are assumed. A) Relationship between cross-immunity (ψ) and contact degree (κ); B) Relationship between ν and κ; C) relationship between μ and κ; D) Relationship between infectious period and κ; E) Relationship between duration of immunity and κ; F) relationship between epidemic cycle and duration of immunity; G) dependence of epidemic cycle and infectious period; and H) dependence of CV on κ. In panels D-H) the blue points represent the parameter values when contact degrees $\kappa \leq \kappa_{SC}$ and the red points those with contact degrees $\kappa > \kappa_{SC}$. The three values above each panel represent the correlation coefficients between the two variables for all the values, the values when $\kappa \leq \kappa_{SC}$, and the values when $\kappa > \kappa_{SC}$.
